# The moderating effect of fluid overload on the relationship between the augmentation index and left ventricular diastolic function in patients with CKD

**DOI:** 10.1038/s41598-023-50746-5

**Published:** 2024-01-04

**Authors:** Byoung-Geun Han, Daewoo Pak, Jae-Seok Kim, Yujin Sohn

**Affiliations:** 1 Department of Nephrology, Yonsei University Wonju College of Medicine, Kang-Won, Wonju, Korea; 2 Division of Data Science, Yonsei University, Kang-Won, Wonju, Korea; 3 Department of Infectious Disease, Yonsei University Wonju College of Medicine, Kang-Won, Wonju, Korea

**Keywords:** Biomarkers, Diseases, Nephrology

## Abstract

Increased vascular stiffness, fluid overload, and left ventricular diastolic dysfunction (LVDD) are common in patients with chronic kidney disease (CKD). We investigated the potential moderating effect of volume status in the relationship between arterial stiffness and left ventricular (LV) diastolic function in non-dialysis patients with stage 5 CKD. The radial augmentation index at a heart rate of 75 beats/min (rAIx75), overhydration/extracellular water (OH/ECW), and E/e´ ratio were concurrently measured in 152 consecutive patients. Each of these parameters reflects the status of vascular stiffness, fluid balance, and LV diastolic function, respectively. Hierarchical regression analysis demonstrated a significant interaction effect of OH/ECW for all patients (*P* = 0.015), even after controlling for confounders. In separate analyses, this interaction effect was particularly significant in women (*P* = 0.010), whereas its significance in patients with diabetes was marginally significant (*P* = 0.062). Our study suggested that fluid overload could be one of the more aggravating factors of LVDD in patients with CKD who have increased arterial stiffness. Therefore, it is advisable to conduct simultaneous assessments of vascular stiffness, fluid balance, and LV function, particularly in the specific groups mentioned earlier. Our results may serve as evidence applicable to patients with chronic heart failure.

## Introduction

Cardiovascular events primarily contribute to morbidity and mortality and are more common in patients with end-stage renal disease (ESRD) than in the general population^1^. Left ventricular diastolic dysfunction (LVDD) is highly prevalent in the general population and individuals with chronic kidney disease (CKD). Patients with severe LVDD have a higher risk of 3-year cardiovascular mortality compared to those without LVDD, even after adjusting for CKD stage and systolic function^2^. The presence of LVDD in patients with CKD, regardless of the underlying cause, is associated with an elevated risk of heart failure with preserved ejection fraction (HFpEF)^3,4^.

Age, diabetes, hypertension, obesity, and ischemic coronary artery disease are established risk factors for developing LVDD in the general population^5–8^. Classical cardiovascular risk factors induce myocardial structural and functional abnormalities through low-grade systemic and endothelial inflammation^9^. However, the pathogenesis of LVDD in CKD is multifactorial and involves a combination of traditional and non-traditional CKD-related risk factors^10^. High blood pressure, as a traditional risk factor, significantly contributes to left ventricular (LV) remodeling in CKD; however, other CKD-related factors, such as chronic inflammation, elevated levels of calcium and phosphorus, uremic toxins, anemia, fluid overload, and vascular calcification are also implicated in the structural and functional changes of the myocardium^11^. The assessment and management of significant factors beyond conventional risk factors for preventing HFpEF in patients with CKD have gained increased importance.

Increased arterial stiffness may be a risk factor for LVDD in patients with hypertension and normotensive subjects^12^. Vascular calcification, which appears as one of the diverse clinical features of chronic kidney disease-mineral bone disorder (CKD-MBD), leads to vascular stiffness, regardless of the underlying cause of CKD^13^. Vascular stiffness has been reported as an independent predictor of all-cause and cardiovascular mortality in patients with ESRD^14^. Notably, there are significant sex differences in the development of arterial stiffness, with women exhibiting higher levels^15^. More importantly, increased arterial stiffness has been associated with a higher risk of mortality in women compared with men and particularly, contributes to the occurrence of HFpEF in women^16^.

Fluid overload may be an independent risk factor for LVDD in patients with non-dialysis CKD^17,18^. In addition to blood pressure regulation, timely identification and effective management of fluid overload, a distinctive feature in patients with advanced-stage CKD, is essential^3^. Notably, most studies, including our previous studies, have evaluated the degree of influence of a single factor on LVDD^17,18^. However, few studies have statistically analyzed the relationship between vascular stiffness, fluid overload, and LV diastolic function concomitantly in a specific group, such as patients with CKD.

Therefore, we investigated fluid overload and its potential interaction effect on the relationship between vascular stiffness and LVDD in dialysis naïve patients with stage 5 CKD (CKD5). Furthermore, we investigated whether this interaction effect differed between men and women.

## Results

### Characteristics of the study patients

The clinical characteristics of the patients based on the E/e´ ratio are presented in Table 1. The patients with diastolic dysfunction (E/e´ ratio > 15) had significantly lower central diastolic blood pressure (cDBP), serum albumin, and calcium levels but higher marker of volume status (overhydration/extracellular water, OH/ECW), central pulse pressure (cPP), left atrial dimension (LAD), left atrial volume index (LAVI), left ventricular mass index (LVMI), and age. The mean ages of the men and women were 59.35 ± 12.53 years and 60.11 ± 15.91 years, respectively. The men accounted for 56.6% (n = 86) of all patients. The radial augmentation index, at a heart rate of 75 beats/min (rAIx75), was significantly higher in the women compared with the men (84.14 ± 15.48 vs. 71.36 ± 12.07; *P* < 0.001). However, there were no statistical differences between the men and women with regards to the E/e´ ratio, LVMI, cPP, cDBP, and OH/ECW (Table 2). Similarly, there were no statistical differences in the E/e´ ratio, LVMI, rAIx75, and age between the patients with and without diabetes. However, significant differences were observed between the patients with and without diabetes in terms of OH/ECW (18.47 ± 14.44% vs. 9.25 ± 12.00%; *P* < 0.001) and cPP (71.36 ± 18.86 mmHg vs. 63.78 ± 19.73 mmHg; *P* = 0.021). In addition, cDBP was significantly lower in the patients with diabetes than those without diabetes (71.23 ± 12.24 mmHg vs. 80.56 ± 16.80 mmHg; *P* = 0.001).

**Table 1:** Comparative analysis of demographics, volume status, echocardiographic parameters, augmentation index, and serum chemistry based on the E/e´ ratio.

Variables	Total (N = 152)	E/e´ ratio		*P*-value
		≤ 15 (n = 93)	> 15 (n = 59)	
Augmentation index at 75	76.91 ± 15.02	75.12 ± 15.60	79.73 ± 13.71 ^a^	0.058
OH/ECW, %	15.23 ± 14.24	12.66 ± 14.67	19.29 ± 12.61	0.005
Age, years	59.68 ± 14.05	56.87 ± 14.10	64.10 ± 12.89	0.002
Sex				
Male	86 (56.6%)	56 (65.1%)	30 (34.9%)	0.256
Female	66 (43.4%)	37 (56.1%)	29 (43.9%)	
Diabetes				
Yes	97 (63.8%)	55 (56.7%)	42 (43.3%)	0.132
No	55 (36.2%)	38 (69.1%)	17 (30.9%)	
cBMI, kg/m^2^	23.81 ± 4.17	24.31 ± 4.64	23.03 ± 3.56	0.065
cSBP, mmHg	142.66 ± 23.11	141.42 ± 23.69	144.63 ± 22.23	0.406
cDBP, mmHg	74.63 ± 14.72	77.76 ± 15.71	69.75 ± 11.54	< 0.001
cPP, mmHg	68.60 ± 19.46	64.58 ± 18.49	74.88 ± 19.43	0.001
LAD, cm	4.53 ± 0.54	4.43 ± 0.53	4.68 ± 0.52	0.005
LAVI, mL/m^2^	40.86 ± 12.45	38.18 ± 11.18	45.08 ± 13.25	0.001
LVEDV, mL	146.76 ± 34.60	144.37 ± 33.61	150.54 ± 36.08	0.285
LVMI, g/m^2^	113.99 ± 28.90	108.57 ± 26.10	122.54 ± 31.32	0.003
LVEF, %	58.43 ± 8.44	59.90 ± 6.31	56.12 ± 10.65	0.016
hs-CRP, mg/dL	0.69 ± 1.86	0.85 ± 2.30	0.45 ± 0.69	0.121
iPTH, pg/mL	330.92 ± 247.56	312.98 ± 210.45	359.67 ± 297.35	0.261
Vitamin D3, ng/mL	15.68 ± 9.82	16.43 ± 10.37	14.50 ± 8.14	0.240
Hemoglobin, g/dL	8.92 ± 1.23	9.06 ± 1.25	8.71 ± 1.18	0.087
Total protein, g/dL	6.01 ± 0.78	6.09 ± 0.81	5.90 ± 0.72	0.152
Albumin, g/dL	3.52 ± 0.54	3.61 ± 0.55	3.38 ± 0.50	0.010
Total Cholesterol, mg/dl	140.59 ± 43.10	140.83 ± 45.05	140.20 ± 39.92	0.931
HDL-C, mg/dL	37.89 ± 12.20	37.28 ± 11.63	38.85 ± 13.07	0.444
LDL-C, mg/dL	77.40 ± 36.32	77.87 ± 37.98	76.68 ± 33.87	0.845
Triglyceride, mg/dl	133.77 ± 61.98	135.15 ± 64.15	131.59 ± 58.88	0.731
Alkaline phosphatase, U/L	82.78 ± 37.05	79.90 ± 30.27	87.32 ± 45.68	0.273
Calcium, mg/dL	7.70 ± 1.09	7.78 ± 1.02	7.31 ± 1.16	0.010
Phosphate, mg/dL	6.16 ± 1.80	5.97 ± 1.02	6.43 ± 1.90	0.124
Uric acid, mg/dL	7.54 ± 2.52	7.43 ± 2.56	7.70 ± 2.46	0.515
eGFR, mL/min/1.73 m^2^	6.61 ± 2.55	6.80 ± 2.70	6.32 ± 2.31	0.264

cBMI, corrected body mass index; cDBP, central diastolic blood pressure; cSBP, central systolic blood pressure; cPP, central pulse pressure; ECW, extracellular water; eGFR, estimated glomerular filtration rate; HDL-C, high-density lipoprotein cholesterol; hs-CRP, high-sensitivity C-reactive protein; iPTH, intact parathyroid hormone; LAD, left atrial dimension; LAVI, left atrial volume index; LDL-C, low-density lipoprotein cholesterol; LVEF, left ventricular ejection fraction; LVEDV, left ventricular end-diastolic volume; LVMI, left ventricular mass index; OH, overhydration.

**Table 2:** Sex differences in demographics, volume status, echocardiographic parameters, augmentation index, and serum chemistry.

Variables	Male (n = 86)	Female (n = 66)	*P*-value
Age, years	59.35 ± 12.53	60.11 ± 15.91	0.751
E/e´ ratio	14.26 ± 5.38	15.71 ± 6.35	0.128
Augmentation index at 75	71.36 ± 12.07	84.14 ± 15.48	< 0.001
OH/ECW, %	16.26 ± 14.04	13.88 ± 14.49	0.308
OH/ECW, %			
≥ 15	43 (63.2%)	25 (36.8%)	0.136
< 15	43 (51.2%)	41 (48.8%)	
Diabetes			
Yes	62 (63.9%)	35 (36.1%)	0.015
No	24 (43.6%)	31 (56.4%)	
cBMI, kg/m^2^	23.74 ± 3.98	23.90 ± 4.43	0.813
cSBP, mmHg	139.63 ± 20.85	146.62 ± 25.39	0.072
cDBP, mmHg	73.31 ± 13.26	76.33 ± 16.36	0.223
cPP, mmHg	67.29 ± 17.66	70.29 ± 21.56	0.362
LAD, cm	4.56 ± 0.50	4.47 ± 0.59	0.319
LAVI, mL/m^2^	40.84 ± 11.05	40.89 ± 14.16	0.978
LVEDV, mL	155.02 ± 36.75	136.00 ± 28.41	0.001
LVMI, g/m^2^	114.60 ± 27.27	113.20 ± 31.09	0.767
LVEF, %	58.14 ± 7.93	58.82 ± 9.10	0.625
hs-CRP, mg/dL	0.92 ± 2.28	0.41 ± 1.07	0.072
iPTH, pg/mL	293.25 ± 153.03	380.75 ± 328.79	0.050
Vitamin D3, ng/mL	16.69 ± 10.24	14.36 ± 9.15	0.147
Hemoglobin, g/dL	9.01 ± 1.12	8.81 ± 1.36	0.319
Total protein, g/dL	5.95 ± 0.76	6.09 ± 0.80	0.280
Albumin, g/dL	3.44 ± 0.55	3.62 ± 0.52	0.044
Total Cholesterol, mg/dl	133.86 ± 40.39	149.35 ± 44.98	0.027
HDL-C, mg/dL	34.44 ± 10.39	42.46 ± 12.96	< 0.001
LDL-C, mg/dL	74.83 ± 35.44	80.82 ± 37.46	0.317
Triglyceride, mg/dl	133.85 ± 63.89	133.67 ± 59.90	0.986
Alkaline phosphatase, U/L	81.73 ± 30.27	84.15 ± 44.58	0.706
Calcium, mg/dL	7.48 ± 1.06	7.75 ± 1.12	0.138
Phosphate, mg/dL	6.30 ± 2.07	5.96 ± 1.37	0.221
Uric acid, mg/dL	7.66 ± 2.45	7.38 ± 2.77	0.494
eGFR, mL/min/1.73 m^2^	7.14 ± 2.77	5.93 ± 2.07	0.004

cBMI, corrected body mass index; cDBP, central diastolic blood pressure; cSBP, central systolic blood pressure; cPP, central pulse pressure; ECW, extracellular water; eGFR, estimated glomerular filtration rate; HDL-C, high-density lipoprotein cholesterol; hs-CRP, high-sensitivity C-reactive protein; iPTH, intact parathyroid hormone; LAD, left atrial dimension; LAVI, left atrial volume index; LDL-C, low-density lipoprotein cholesterol; LVEF, left ventricular ejection fraction; LVEDV, left ventricular end-diastolic volume; LVMI, left ventricular mass index; OH, overhydration.

### Correlation of volume status, echocardiographic findings, and serum chemistry with the E/e´ ratio

In all patients, the E/e´ ratio was positively associated with various factors, including age, rAIx75, cPP, LAD, LAVI, left ventricular end diastolic volume (LVEDV), LVMI, and OH/ECW. However, cDBP, LV ejection fraction (LVEF), hemoglobin, total protein, albumin, and serum calcium levels were negatively associated (Supplementary Table S1).

### Moderating effect of OH/ECW

In order to analyze the moderating effect, both the predictor and moderator need to have significant associations with the dependent variable. However, there should be no significant correlation between the predictor and the moderator. These are common prerequisites for conducting a moderation analysis. Our study showed no association between the predictor (rAIx75) and the moderator (OH/ECW) (*P* = 0.992). However, rAIx75 and OH/ECW are significantly associated with the E/e´ ratio. In stepwise multiple linear regression analyses, sex, LVMI, LVEF, cDBP, cPP, and serum albumin levels were shown to be substantially associated with the E/e´ ratio. The interaction effect of OH/ECW for all the patients was statistically significant even after adjusting for age, sex, diabetes prevalence, LVMI, LVEF, cDBP, cPP, and serum albumin levels (*P* = 0.015) (Table 3). However, the subgroup analyses conducted based on sex and diabetes did not yield consistent findings regarding the moderating effect of OH/ECW on the E/e´ ratio (Tables 4 and 5). In the separate analyses conducted by sex and adjusting for age, diabetes prevalence, LVMI, LVEF, cDBP, cPP, and serum albumin levels, the interaction effect was significant only in women (P = 0.010). Interestingly, the interaction effect of OH/ECW after adjusting for age, sex, LVMI, LVEF, cDBP, cPP, and serum albumin levels for patients with diabetes was marginally significant (*P* = 0.062).

**Table 3:** Outcomes from hierarchical moderated regression analysis examining the moderating effects of OH/ECW on the E/e´ ratio for all patients.

Variables	E/e´ ratio				
	Adjusted R^2^	B	SE	*Beta*	*P*-value
Covariates	0.406				
Age		0.072	0.038	0.172	0.063
Sex		2.113	0.868	0.179	0.016
Diabetes prevalence		0.648	0.879	0.053	0.462
LVMI		0.067	0.015	0.331	< 0.001
LVEF		− 0.179	0.048	− 0.259	< 0.001
cDBP		− 0.052	0.034	− 0.132	0.127
cPP		0.018	0.030	0.060	0.542
Albumin		− 1.595	0.962	− 0.147	0.100
Main effects					
rAIx75	0.402	0.001	0.038	0.002	0.986
OH/ECW	0.402	0.030	0.039	0.073	0.442
Interaction					
rAIx75 x OH/ECW	0.423	0.005	0.002	0.161	0.015

cDBP, central diastolic blood pressure; cPP, central pulse pressure; ECW, extracellular water; LVEF, left ventricular ejection fraction; LVMI, left ventricular mass index; rAIx75, radial augmentation index at 75; OH, overhydration; SE, standard error.

**Table 4:** Results of hierarchical moderated regression analyses for men and women.

Subgroup	Variables	E/e´ ratio				
		Adjusted R^2^	B	SE	*Beta*	*P*-value
Men	*Covariates*	0.282				
	Age		0.066	0.059	0.151	0.269
	Diabetes prevalence		1.075	1.386	0.090	0.441
	LVMI		0.061	0.023	0.308	0.010
	LVEF		− 0.154	0.073	− 0.227	0.039
	cDBP		− 0.039	0.054	− 0.096	0.468
	cPP		0.031	0.045	0.100	0.499
	Albumin		− 1.948	1.436	− 0.197	0.179
	*Main effects*					
	rAIx75	0.276	− 0.036	0.064	− 0.081	0.574
	OH/ECW	0.267	− 0.018	0.064	− 0.047	0.780
	*Interaction*					
	rAIx75 x OH/ECW	0.257	− 0.001	0.004	− 0.016	0.883
Women	*Covariates*	0.471				
	Age		0.094	0.055	0.237	0.093
	Diabetes prevalence		0.660	1.281	0.052	0.608
	LVMI		0.070	0.022	0.342	0.002
	LVEF		− 0.229	0.069	− 0.328	0.002
	cDBP		− 0.057	0.046	− 0.146	0.228
	cPP		0.018	0.042	0.062	0.668
	Albumin		− 0.949	1.408	− 0.077	0.503
	*Main effects*					
	rAIx75	0.461	0.003	0.049	0.006	0.957
	OH/ECW	0.472	0.064	0.055	0.145	0.248
	*Interaction*					
	rAIx75 x OH/ECW	0.524	0.007	0.003	0.237	0.010

cDBP, central diastolic blood pressure; cPP, central pulse pressure; ECW, extracellular water; LVEF, left ventricular ejection fraction; LVMI, left ventricular mass index; rAIx75, radial augmentation index at 75; OH, overhydration; SE, standard error.

**Table 5:** Results of hierarchical moderated regression analyses for patients with and without diabetes.

Subgroup	Variables	E/e´ ratio				
		Adjusted R^2^	B	SE	*Beta*	*P*-value
Patients without diabetes	*Covariates*	0.588				
	Age		− 0.014	0.057	− 0.037	0.807
	Sex		1.912	1.282	0.144	0.143
	LVMI		0.062	0.021	0.300	0.005
	LVEF		− 0.201	0.077	− 0.240	0.013
	cDBP		− 0.037	0.052	− 0.095	0.475
	cPP		0.090	0.056	0.266	0.117
	Albumin		− 2.831	1.607	− 0.194	0.085
	*Main effects*					
	rAIx75	0.582	− 0.041	0.060	− 0.098	0.503
	OH/ECW	0.608	0.128	0.067	0.232	0.062
	*Interaction*					
	rAIx75 x OH/ECW	0.613	0.003	0.003	0.117	0.210
Patients with diabetes	*Covariates*	0.231				
	Age		0.090	0.057	0.197	0.120
	Sex		2.082	1.228	0.190	0.094
	LVMI		0.064	0.022	0.325	0.004
	LVEF		− 0.160	0.063	− 0.264	0.013
	cDBP		− 0.036	0.047	− 0.083	0.441
	cPP		− 0.006	0.037	− 0.023	0.862
	Albumin		− 1.718	1.248	− 0.182	0.172
	*Main effects*					
	rAIx75	0.223	0.018	0.053	0.051	0.730
	OH/ECW	0.215	− 0.003	0.051	− 0.008	0.954
	*Interaction*					
	rAIx75 x OH/ECW	0.237	0.005	0.003	0.193	0.062

cDBP, central diastolic blood pressure; cPP, central pulse pressure; ECW, extracellular water; LVEF, left ventricular ejection fraction; LVMI, left ventricular mass index; rAIx75, radial augmentation index at 75; OH, overhydration; SE, standard error.

## Discussion

The LVDD in pre-dialysis patients with CKD is associated with poor cardiovascular outcomes and increased all-cause mortality rates^19,20^. Among the known risk factors for LVDD, we evaluated the impact of the relationship between vascular stiffness and fluid overload on LVDD in patients with advanced-stage CKD^21^. The factors that are more strongly associated with LVDD may vary depending on the stages of CKD; however, vascular stiffness and fluid overload have been reported as significantly associated with the development of LVDD as kidney function declines.

CKD has been proposed as a model of early vascular aging since it is associated with arterial stiffness; this plays a role in the development of cardiovascular disease (CVD)^22,23^. In CKD, multifactorial pathophysiological mechanisms contribute to arterial remodeling and premature vascular aging, ultimately resulting in arterial hardening. Vascular calcification, especially as a part of CKD-MBD in patients with CKD or ESRD, is recognized as one of the main factors contributing to vascular stiffening^24^. The augmentation index (AIx), which represents the functional status of the central and peripheral arteries, is reported to be an independent predictor of all-cause and cardiovascular mortality in patients with ESRD^14^. This study evaluated vascular stiffness using rAIx75 derived from pressure waveform analysis.

Notably, several studies have investigated whether the association between arterial stiffness and echocardiographic markers of LVDD was significant in patients with various diseases and in the general population^12,16,25,26^. However, studies targeting patients with CKD are rare. Declining kidney function induces structural and functional vascular changes, significantly affecting central and peripheral hemodynamics. In CKD, uncontrolled fluid overload also leads to changes in the hemodynamics. Furthermore, all the factors mentioned above are ultimately associated with structural and functional changes in the myocardium. However, as mentioned earlier, there have been few attempts to analyze vascular stiffness, fluid overload, and LVDD within a single framework.

It is well known that arterial stiffening increases with advancing age^27^. With aging or in diseases, such as CKD, that promote vascular calcification and arterial stiffness, the central arteries become stiff earlier than the brachial arteries; this results in a larger forward wave amplitude, earlier arrival of reflected waves, and increased pulse pressure^28^. Consequently, cPP increases more and earlier than peripheral pulse pressure. That is why we used central blood pressure only as a covariate and not peripheral blood pressure. Besides age, several clinical factors have been reported as risk factors for vascular stiffening. In our study, other known risk factors were also as covariates. Factors associated with the occurrence of CKD-MBD and vascular calcification, such as vitamin D3, high-sensitivity C-reactive protein, and intact parathyroid hormone, showed no statistical significance in t-tests and Pearson correlations. Additionally, they did not yield statistical significance in stepwise linear regression analyses aimed at identifying potential determinants of LV diastolic function. As a result, these factors were not employed as covariates in our study.

In the general population, left ventricular hypertrophy (LVH) has been reported as one of the pathogenetic mechanisms of LVDD. In patients with CKD, both LVDD and LVH are common and closely related^29^. Although it remains unclear whether cardiac structural changes precede functional changes in CKD or vice versa, LVMI was also included as one of the confounders in this analysis. In patients with diabetes, structural and functional alterations occur in the cardiac tissue, leading to impaired LV diastolic function. Therefore, diabetes is independently associated with impaired LV diastolic function^6^. Furthermore, arterial stiffness and fluid imbalance contribute to LVDD in patients with diabetes and CKD^17,25^. Herein, diabetes was used as a covariate for all patients, and the differences in the interaction effect between the presence or absence of diabetes were investigated as a subgroup analysis.

Vascular stiffening associated with aging exhibits sexual dimorphism, with young women having lower stiffness than age-matched men. However, this sex difference reverses during normal aging^30^. Notably, numerous studies have previously reported the sex differences in the arterial changes associated with vascular stiffness^15,31^. Investigations have also explored the sex-specific association between arterial stiffness and LVDD, showing a significant correlation between LVDD and an arterial stiffness biomarker in women but not in men^26^. Sex-specific differences in long-term outcomes of HFpEF were observed, with ventricular-vascular stiffening being significant in women and heart rate playing a significant role in men^32^. These findings suggested that higher arterial stiffness in women may increase their susceptibility to developing HFpEF^4,16^. Patients with HFpEF and fluid overload generally experience worse prognoses than those with normal fluid volume^33^. Increased vascular stiffness and fluid overload may have coincidentally occurred together due to the nature of CKD, however, there are few studies simultaneously evaluating the interaction with the LVDD biomarker. In this study, we observed that rAIx75 and OH/ECW each had a positive correlation with the E/e´ ratio. Furthermore, a significant interaction between rAIx75 and OH/ECW was identified across all the patient groups. The positive B-coefficient for the interaction term, which was evident in all the patients, women, and the diabetes subgroup, indicated a tendency for the E/e´ ratio to increase gradually with elevated vascular stiffness and higher OH/ECW values. Interestingly, the results suggested a gradual deterioration of the diastolic function as arterial stiffness rises and fluid overload increases, which was particularly notable in women. The factor causing the differences between men and women does not appear to be fluid overload, but rather seems to be influenced by differences in vascular stiffness. Subsequently, we also consider that there is a difference in the interaction effect of OH/ECW. Additionally, the interaction between rAIx75 and OH/ECW remained marginally significant in the diabetes group after covariate correction, but not in the non-diabetic group. Our study suggested a potential vulnerability to the occurrence of LVDD or HFpEF, particularly in postmenopausal or elderly women with advanced-stage CKD. However, given that our study primarily comprised older women, conducting an analysis that stratifies between menstruating and postmenopausal women could potentially yield different results.

This study has several limitations. First, it should be noted that the formal definition of LVDD involves additional echocardiographic measurements beyond the E/e´ ratio^34^. However, in line with previous studies, a simplified and modified definition of LVDD (specifically, E/e´ ratio > 15) was used in this study. Second, the rAIx75 was employed as an indicator to assess arterial stiffness, whereas the aortic AIx was omitted. However, the rAIx75 demonstrated a robust correlation with aortic AIx^35^. Third, we did not simultaneously assess the severity of vascular calcification, which is directly and closely associated with aortic stiffness, using other evaluation methods, such as quantitative computerized tomography. Moreover, while sex-specific risk factors, including hormonal changes, have been suggested to contribute to the susceptibility to the development of LVDD and subsequent heart failure^36^, we did not assess the role of hormone variations. Therefore, at this point, generalizing our results to other early-stage patients, especially young women, is limited. Despite these limitations, the strengths of our study include that we conducted our study on a relatively homogenous group of patients with CKD5, based on the estimated glomerular filtration rate. Additionally, the assessment of vascular stiffness and volume status was simultaneously and objectively measured at the time of echocardiography. To the best of our knowledge, this is the first study to have attempted such analysis.

Considering these results, it is worth noting that fluid overload could be one of the aggravating factors of LVDD in patients with CKD5 who have increased arterial stiffness. It is advisable to perform simultaneous assessments of vascular stiffness, fluid balance, and LV function, particularly in the specific groups mentioned earlier (women and patients with diabetes). Therefore, implementing tailored treatment strategies targeting this risk factor may prevent cardiac structural and functional impairments. Furthermore, these findings may serve as evidence applicable to patients with chronic heart failure.

## Materials and methods

### Patients and data collection

Since 2014, we have registered consecutive patients with CKD5 into the bio-impedance spectroscopy (BIS) cohort after receiving approval from the Institutional Review Board of Yonsei University Wonju Severance Christian Hospital. As for our BIS cohort, various tests including bio-impedance measurement, echocardiography, and laboratory evaluations prior to the initiation of dialysis treatment were basically performed when patients with CKD5 who needed initiation of renal replacement therapy were admitted to a planned hospitalization. Patients underwent additional necessary tests based on their systemic condition and underwent further examinations as advised by other departments. Of the patient cohorts, 152 patients were simultaneously evaluated for their central blood pressure, pulse pressure, and AIx obtained from the radial arterial waveform. This study is the result of separately selecting and analyzing only patients who measured AIx at the same time in the process of analyzing the data of patients participating in the cohort. Therefore, the current study was a retrospective observational study . Among the analyzed patients, there were individuals with clinically insignificant conditions like first-degree atrioventricular block. However, there were no patients with arrhythmias, such as atrial fibrillation, that could potentially affect cardiac function. This study was conducted in accordance with the Declaration of Helsinki. All patients provided written informed consent before participating in the study.

### Conventional echocardiographic study

Comprehensive echocardiographic measurements were conducted using a 3-MHz transducer and a commercial ultrasound system (GE Vivid E9; GE Healthcare, Chicago, IL, USA). The imaging procedures followed standard techniques, including M-mode, two-dimensional, and Doppler measurements, as recommended by the American Society of Echocardiography and the European Association of Cardiovascular Imaging guidelines^34,37^. Transmitral inflow velocities were assessed using pulsed-wave Doppler in the apical four-chamber view, with the sample volume placed at the tips of the mitral valve leaflet. Early diastolic (E-wave) velocities were measured. Tissue Doppler imaging in the apical four-chamber view was used to measure the LV myocardial velocities, with the sample volume placed at the septal mitral annulus. The peak early (e′) diastolic mitral annular velocity was measured, and the E/e′ ratio was calculated. An E/e′ ratio > 15 commonly indicates elevated LV filling pressure, and this cut-off value was used to determine the presence or absence of LVDD. The LAD was measured using 2D-guided M-mode echocardiography in the parasternal short-axis view at the base of the heart. The left atrial (LA) volume was computed using the area-length approximation formula: LA volume (mL) = [8/(3π)][(A1 × A2)/L], where A1 and A2 represent the corresponding LA areas measured in the apical two- and four-chamber views. The LA length is the shortest of the two long axes measured in each view. The LAVI was calculated by dividing LA volume by body surface area (BSA), which was determined using the following formula: BSA (m^2^) = 0.007184 × weight^0^^.425^ × height^0.725^. The LV mass was calculated using the following equation: LV mass (g) = 0.8 × {1.04 × ([IVS + LVID + PWT]^3^ − [LVID]^3^)} + 0.6, where IVS represents the interventricular septum, LVID is the LV internal diameter, and PWT denotes the inferolateral wall thickness. All measurements were obtained at end-diastole. To correct for BSA, the LVMI was calculated by dividing LV mass by BSA. Following the previously mentioned recommendations, LVEDV and LVEF were measured using the biplane modified Simpson’s rule. Trained cardiologists, blinded to the patients’ clinical information, performed the echocardiography.

### Assessment of the volume status

Whole-body BIS was performed using BCM (Body Composition Monitoring™, Fresenius Medical Care AG & Co., Bad Homburg vor der Höhe, Germany) to assess body fluid balance. Extracellular water (ECW), intracellular water (ICW), and total body water (TBW) were automatically provided by onboard software of the BCM device using equations based on the Hanai mixture theory developed by Moissl et al^38^. A three-compartment BIS model separates body weight into normally hydrated lean tissue mass, normally hydrated adipose tissue mass, and extracellular fluid overload, commonly represented as the volume of overhydration (OH)^39^. OH, presented as a positive or negative value, can be calculated from the difference between the actual measured ECW and the normally expected ECW^40^. Validation was performed against bromide*/*deuterium dilution for ECW*/*TBW and total body potassium for ICW. A good agreement with reference methods based on tracer dilution was achieved. The accuracy and precision of BCM for fluid volumes was reported as − 0.4 ± 1.4 L (mean ± SD) for ECW, 0.2 ± 2.0 L for ICW and − 0.2 ± 2.3 L for TBW. The reference data of OH from the 1247 healthy subjects showed that normohydration is defined by a range (− 1.1 to 1.1 L)^41^. The 10th and 90th percentiles of the healthy subjects yielded to − 1.1 L and + 1.1 L, respectively. In this study, since the clinical significance of OH values vary based on the patient body size, relative overhydration (OH/ECW) was primarily used to determine the volume status for the analysis. The body mass index (BMI) of patients was recalculated as the corrected BMI (cBMI) by adjusting for fluid overload using the formula: cBMI (kg/m^2^) = (Body weight − OH)/height^2^.

### Estimation of central blood pressure

The central blood pressure, cPP, and AIx were noninvasively obtained from the radial arterial waveform using an arterial applanation tonometry method (HEM-9000AI; Omron Matsusaka Co., Ltd., Matsusaka, Japan). The augmentation of central blood pressure is a manifestation of early wave reflection when the reflected wave arrives at the central aorta. The AIx was defined as the increment in pressure from the first systolic shoulder (inflection point) to the systolic pressure peak. The radial AIx (rAIx) values are automatically calculated as follows: (SP2–bDBP)/(SP1–bDBP) × 100 (%), where SP1 and SP2 represent the first and second peaks of the peripheral systolic pressure, and bDBP denotes the brachial diastolic blood pressure. The rAIx has a strong correlation with aortic AIx^35,42^. All measurements were conducted following the procedures described in the previous studies^43^. According to Wilkinson et al*.*, the AIx is influenced inversely and linearly by the heart rate; hence, it was normalized at a heart rate of 75 beats per min.

### Statistical analysis

The demographic and clinical characteristics of the patients were summarized using means and standard deviations for continuous variables and frequencies and percentages for categorical variables. Based on their E/e´ ratio, the patients were divided into E/e´ ≤ 15 and E/e´ > 15, indicating the absence or presence of LVDD. Furthermore, we conducted a comparative analysis of volume status, echocardiographic parameters, AIx, and serum chemistry between male and female. Differences in clinical variables between the two groups were tested using a chi-squared test and a two-sample t-test, whichever was appropriate. The Pearson correlation coefficients were calculated to evaluate the associations between the E/e´ ratio and other variables, including laboratory findings, echocardiographic parameters, and markers of volume status. We first performed stepwise linear regression analyses to identify the potential determinants of the LV diastolic function. The parameters significantly associated with the E/e´ ratio (*P* < 0.05) were used preferentially in the moderating effect analyses. We conducted a hierarchical regression analysis to examine the moderating effect of OH/ECW on the relationship between the rAIx75 and E/e´ ratio, wherein E/e´ ratio was a dependent variable. The interaction term between rAIx75 and OH/ECW and covariates such as age, sex, diabetes prevalence, LVMI, LVEF, cDBP, cPP, and serum albumin levels were considered as a block being entered in the step of the regression analysis. Mean centering of the rAIx75 and OH/ECW was performed prior to computing the product interaction term. Furthermore, additional analyses were conducted using the same methodology to investigate the differences among the subgroups based on sex and the presence of diabetes, adjusted for aforementioned confounders, as appropriate. Statistical analyses were performed using the IBM SPSS Statistics software (version 25.0; IBM, Armonk, NY, USA). Statistical significance was set at *P* < 0.05.

### Supplementary Information

Supplementary Table S1.

## Data Availability

The datasets generated and/or analysed during the current study are not publicly available due to ethical concerns, as it is not possible to anonymise data sufficient for public access. However, data is available on reasonable request to the corresponding author or the institutional review board of our hospital (irb@yonsei.ac.kr).
