# Supplementary material for: The moderating effect of fluid overload on the relationship between the augmentation index and left ventricular diastolic function in patients with CKD
Source: Sci Rep. 2024 Jan 4;14:480. doi: 10.1038/s41598-023-50746-5 (PMC10767097; doi:10.1038/s41598-023-50746-5)
Supplement: Supplementary file 1 — Supplementary Table S1. [file 41598_2023_50746_MOESM1_ESM.docx]

Supplementary Table S1. Correlation of the E/e´ ratio with other variables.

| Variables | E/e´ ratio | |
| --- | --- | --- |
|  | Correlation coefficient | P-value |
| Augmentation index at 75 | 0.201 | 0.013 |
| OH/ECW, % | 0.300 | <0.001 |
| Age, years | 0.298 | <0.001 |
| cBMI, kg/m^2^ | -0.143 | 0.080 |
| cSBP, mmHg | 0.145 | 0.074 |
| cDBP, mmHg | -0.251 | 0.002 |
| cPP, mmHg | 0.337 | <0.001 |
| LAD, cm | 0.376 | <0.001 |
| LAVI, mL/m^2^ | 0.400 | <0.001 |
| LVEDV, mL | 0.262 | 0.001 |
| LVMI, g/m^2^ | 0.464 | <0.001 |
| LVEF, % | -0.354 | <0.001 |
| hs-CRP, mg/dL | -0.054 | 0.514 |
| iPTH, pg/mL | 0.004 | 0.958 |
| Vitamin D3, ng/mL | -0.160 | 0.049 |
| Hemoglobin, g/dL | -0.175 | 0.031 |
| Total protein, g/dL | -0.185 | 0.022 |
| Albumin, g/dL | -0.264 | 0.001 |
| Total Cholesterol, mg/dl | 0.122 | 0.134 |
| HDL-C, mg/dL | 0.037 | 0.651 |
| LDL-C, mg/dL | 0.117 | 0.153 |
| Triglyceride, mg/dl | 0.035 | 0.672 |
| Alkaline phosphatase, U/L | -0.031 | 0.709 |
| Calcium, mg/dL | -0.231 | 0.004 |
| Phosphate, mg/dL | 0.101 | 0.217 |
| Uric acid, mg/dL | 0.106 | 0.196 |
| eGFR, mL/min/1.73 m^2^ | -0.136 | 0.095 |

cBMI, corrected body mass index; cDBP, central diastolic blood pressure; cSBP, central systolic blood pressure; cPP, central pulse pressure; ECW, extracellular water; eGFR, estimated glomerular filtration rate; HDL-C, high-density lipoprotein cholesterol; hs-CRP, high-sensitivity C-reactive protein; iPTH, intact parathyroid hormone; LAD, left atrial dimension; LAVI, left atrial volume index; LDL-C, low-density lipoprotein cholesterol; LVEF, left ventricular ejection fraction; LVEDV, left ventricular end-diastolic volume; LVMI, left ventricular mass index; OH, overhydration.
